# The Photovoltaic Cell Based on CIGS: Principles and Technologies

**DOI:** 10.3390/ma15051908

**Published:** 2022-03-04

**Authors:** Billel Salhi

**Affiliations:** Interdisciplinary Research Center for Membranes and Water Security, King Fahd University of Petroleum and Minerals, Dhahran 31261, Saudi Arabia; billel@kfupm.edu.sa

**Keywords:** solar cells, CIGS, energy harvesting

## Abstract

Semiconductors used in the manufacture of solar cells are the subject of extensive research. Currently, silicon is the most commonly used material for photovoltaic cells, representing more than 80% of the global production. However, due to its very energy-intensive and costly production method, other materials appear to be preferable over silicon, including the chalcopyrite-structured semiconductors of the CIS-based family (Cu(In, Ga, Al) (Se, S)_2_). Indeed, these compounds have bandwidths between 1 eV (CuInSe_2_) and 3 eV (CuAlS_2_), allowing them to absorb most solar radiation. Moreover, these materials are currently the ones that make it possible to achieve the highest photovoltaic conversion efficiencies from thin-film devices, particularly Cu(In, Ga)Se_2_, which is considered the most efficient among all drifts based on CIS. In this review, we focus on the CIGS-based solar cells by exploring the different layers and showing the recent progress and challenges.

## 1. Introduction

For several years, the field of photovoltaics has piqued the interest of academic researchers and industry. Despite its intermittent nature, it is a very promising technology due to its geographical accessibility and inexhaustible reserves. Its development, which is more important than ever, is aided by political initiatives such as Horizon 2020, which aims to increase energy efficiency in the European Union by 2020, with 20% renewable energy. The photovoltaics market is dominated by the first generation of crystalline silicon solar cells. Other semiconductor materials are present with the second generation of thin-film photovoltaic cells. The materials in this second generation of devices have a significant absorption coefficient. They require a thickness of only a few microns, in comparison to approximately 200 microns required for silicon to absorb incident light sufficiently. This reduction in thickness allows material and cost savings. Thin-film technologies are essential for certain specific applications (flexible substrate, large surface area, semi-transparency, etc.). Previously reserved for roofs, integrating a photovoltaic function with glazing appears to be a preferred path for thin-film PV technologies. With the very significant recent progress in the field of Cu(In, Ga)Se (CIGS), several characteristics have been attributed to photovoltaic cells: high photovoltaic efficiency, stability of performance, and a low-cost industrial manufacturing method. Various methods make it possible to obtain the active absorbing layer in CIGS: co-evaporation (the technique giving the best current photovoltaic yields: 22.6% record value of ZSW [1]), electro-deposition, and magnetron cathode sputtering. This latter technique is generally compatible with the industrial mass production of thin films on a large surface. It also allows the removal of the high-temperature selenization step, a limiting point in some applications. CIGS-based photovoltaic cells consist of a stack of thin layers deposited on a glass substrate: a lower molybdenum (Mo) electrode, a CIGS absorbing layer, a CdS buffer layer, and an upper oxide electrode, namely zinc-doped aluminum (ZnO: Al). Co-evaporation and the CdS buffer layer deposit the CIGS active layer by a chemical bath in the standard process. Since these deposition methods are incompatible with large areas, we chose to create all the cell layers using only the magnetron sputtering technique.

## 2. Photovoltaic Cell Based on CIGS

### 2.1. The Usual Materials

**Figure 1:**
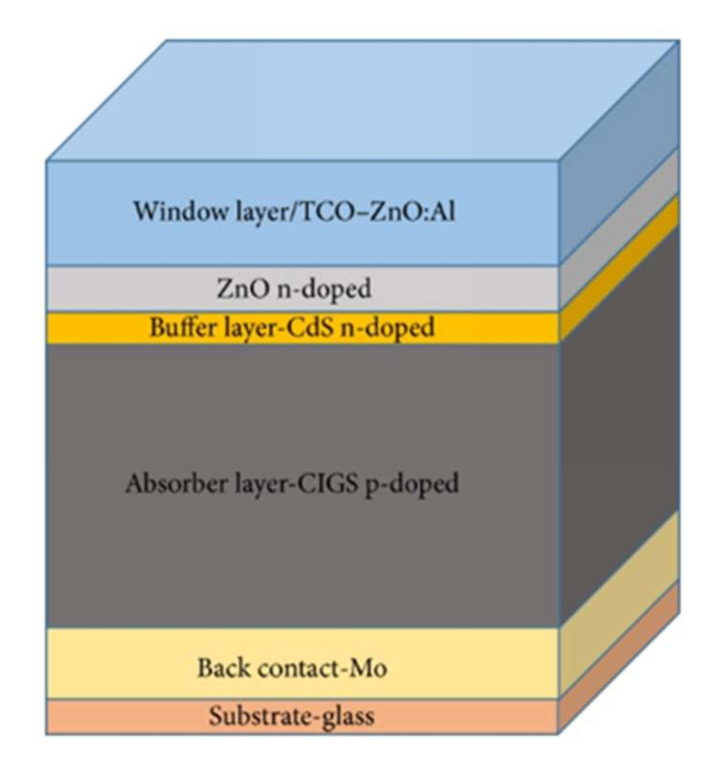
Representation of the standard stack of a CIGS-based solar cell.

The photovoltaic cell based on CIGS consists of a stack of several thin layers of different materials deposited on a substrate (Figure 1).

Each material has a particular function. Starting from the bottom of the cell, there is the substrate, the rear contact, the absorber, the buffer layer, and the window layer.

#### 2.1.1. The Substrate

The substrate generally used is soda–lime glass (or SLG). It must be insulating and stable in order to resist successive deposits. It has a coefficient of thermal expansion close to that of the CIGS, which limits the generation of thermal stresses during the production of films [2]. Other materials, such as steel or flexible substrates, are also used [3,4]. Glass has the advantage of spontaneously supplying the necessary sodium for the excellent performance of the cell [5]. To control the quantity of sodium brought to the cell, studies propose to deposit a barrier to the diffusion of the sodium on the glass and then bring the sodium by an external strategy [6,7].

#### 2.1.2. Molybdenum Rear Contact

The molybdenum film is deposited on the glass substrate with a thickness of approximately 500 nm by sputtering [8]. This film has the function of collecting the carriers; it is the back electrode. Molybdenum is the most promising material for this function because, during the growth of CIGS, an interfacial layer of MoSe_2_ is formed between Mo and CIGS [9] and constitutes an excellent ohmic contact. In addition, the Mo layer and its volume morphology allow the migration of sodium to the absorber from the glass substrate [7].

#### 2.1.3. The Absorber in CIGS

The material Cu(In, Ga)Se_2_ or CIGS is the cell’s absorber. Its chemical composition and properties vary according to the substitution rate between indium and gallium, from CuInSe_2_ to CuGaSe_2_. This is a p-type semiconductor material. Its intrinsic doping is ensured by the formation of defects such as copper gaps, and the substitution between the indium and gallium elements brings about the variation in the gap. CIGS is the semiconductor that is best suited for absorbing solar radiation, with a direct energy gap of between 1.014 eV (CuInSe_2_) and 1.697 eV (CuGaSe_2_) [10]. Additionally, this material has a high absorption coefficient of greater than 10^5^ cm^−1^, which enables its use in thin layers, and a broad spectrum of absorption for thicknesses of the order of 2 μm. Generally, the composition chosen for the cells corresponds to a Ga/(Ga + In) ratio ≈ 0.3, which corresponds to a gap in the range of (1.1–1.2 eV).

Moreover, the diffusion of doping elements such as sodium or potassium from the glass substrate contributes to extrinsic doping. Several filing techniques can perform the filing of CIGS. The most used are co-evaporation, sputtering, and electroplating [10]. Co-evaporation corresponds to the evaporation of Cu, In, and Ga metal elements under a selenium atmosphere [11]. This is the historical technique that has achieved the first world record in yield and whose current yields remain among the best. Sputtering, the principle of which will be detailed in Section 2, corresponds to the sputtering of a target, which is the solid precursor in a neutral atmosphere or reactive with gas H_2_S or H_2_Se [12,13]. These first two deposit techniques are processes that take place under a vacuum. The electrolytic deposition corresponds to the electrodeposition of the metallic elements from a chemical bath containing all the species to be deposited, followed by an annealing step under a selenated or sulfurated atmosphere [14].

#### 2.1.4. The Buffer Layer in CdS

The buffer layer is generally composed of cadmium sulfide (CdS), which constitutes the n-type semiconductor in the p–n heterojunction formed between the CIGS and the CdS, a junction allowing the separation of the charge carriers. This layer of CdS also has the property of passivating the surface defects of the absorber [15]. As a standard, it is deposited by a chemical bath. Since cadmium is a toxic element, other materials are being studied for replacement. We can cite Zn(O,S) [16] and InS [17], which are most often reported in the literature. 

#### 2.1.5. The Intrinsic Zinc Oxide/Zinc Oxide Aluminum-Doped Window Layer (iZnO/ZnO: Al)

The role of the window layer (upper electrode) is to collect the carriers. It generally consists of a stack of two materials: intrinsic zinc oxide and aluminum-doped zinc oxide, which are deposited by sputtering. The intrinsic zinc oxide (n-type semiconductor) is deposited with a small thickness of approximately 50 nm. Its function is to limit electronic losses [18]. On the other hand, aluminum-doped zinc oxide (AZO) is deposited with a greater thickness (from 200 to 400 nm) and is used to collect carriers. The AZO material is a transparent conductive oxide (OTC).

### 2.2. CIGS Cell Band Structure

**Figure 2:**
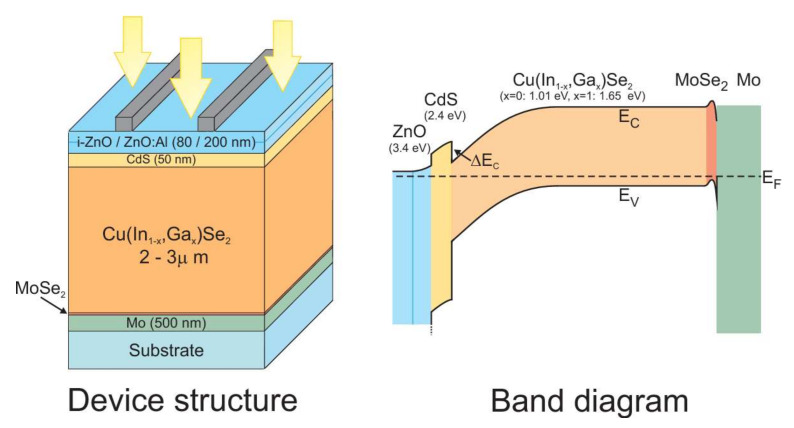
Illustration of the CIGS device structure (**left**) and the corresponding band diagram (**right**). The bandgap of the different materials is also indicated [19]. In Copyright-Non-Commercial Use Permitted.

The band structure of a CIGS-based photovoltaic cell is shown in Figure 2 [19]. After absorption of the light radiation, the creation of the electron–hole pairs take place within the absorber material. The molybdenum rear contact collects the holes. The formation of a MoSe layer at the CIGS/Mo interface constitutes the ohmic contact between the absorber and the back contact. It generates a band curvature for efficiently collecting the holes and pushing the electrons back to the upper electrode.

In order to avoid volume and interface recombinations, it is necessary to remove the electronic junction of the physical junction (CdS/CIGS) rich in defects. This is why the p–n junction must be pushed back (buried) into the CIGS volume. To do this, in a standard manner, a surface inversion is performed by generating a gallium gradient on the surface of the absorber. Depending on the quantity of gallium, the CIGS can be a p-type or n-type semiconductor.

The junction between the CdS and the CIGS thus constitutes an energy barrier at the conduction band level. This barrier is favorable to the proper functioning of the cell because it increases the inversion of the absorber at the surface with the creation of an n-type zone. The front contact in ZnO then collects the electrons.

### 2.3. The Buffer Layer

Regardless of the material and method of deposit used, the buffer layer must fulfill many functions, which we present in this section. Therefore, after recalling the limitations of the CdS material, we will show that the ZnO_x_S_1−x_ and its properties make it possible to meet the different prerequisites.

#### 2.3.1. The Role of the Buffer Layer

**Figure 3:**
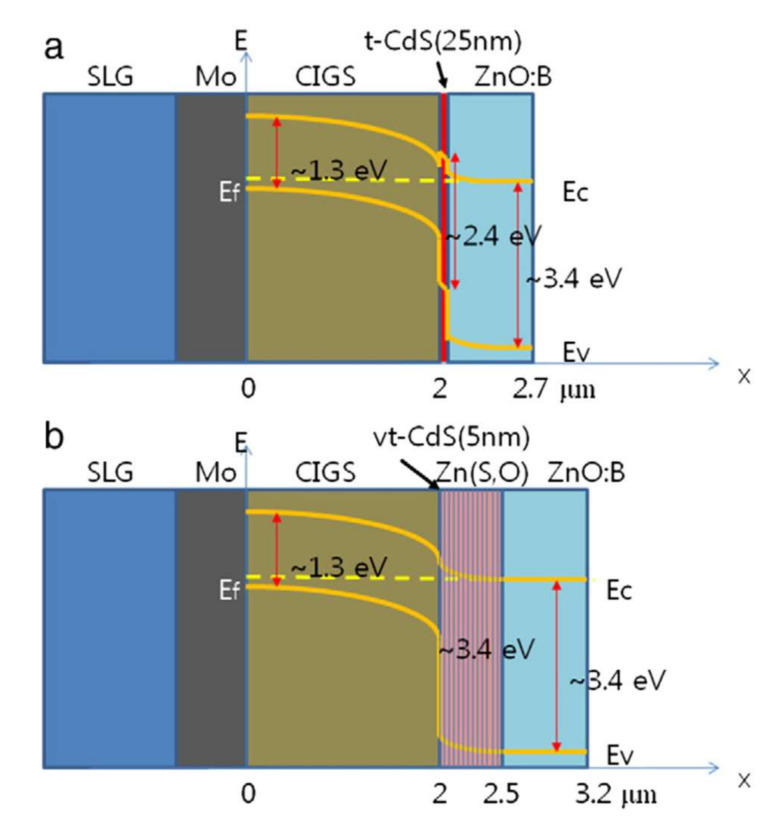
(**a**) Structure of a CIGS/t-CdS/ZnO:B solar cell with a schematic energy diagram, and (**b**) Structure of a CIGS/vt-CdS/Zn(S,O)/ZnO:B solar cell with a schematic energy diagram. Reprinted with permission from Ref. [20]. Copyright 2012 Elsevier.

The primary role of the buffer layer, an n-type semiconductor material, is to form the p–n junction with the p-type CIGS absorber. It usually consists of CdS. The buffer layer plays an important role in the alignment of the conduction bands between the absorber and the window layer. The band structure of the heterojunction between the CIGS and the CdS has a positive conductive band offset (CBO). The band structure of a standard CIGS cell (Figure 3) illustrates this discontinuity generated by the difference between bandgap energies. The different electronic affinities of the two heterojunction materials and the continuity with the vacuum level lead to the energy scheme illustrated below. The CBO value corresponds to the difference in electronic affinity. The value of valence band offset (VBO) is associated with the difference in bandwidths, from which the value of the CBO is subtracted.

**Figure 4:**
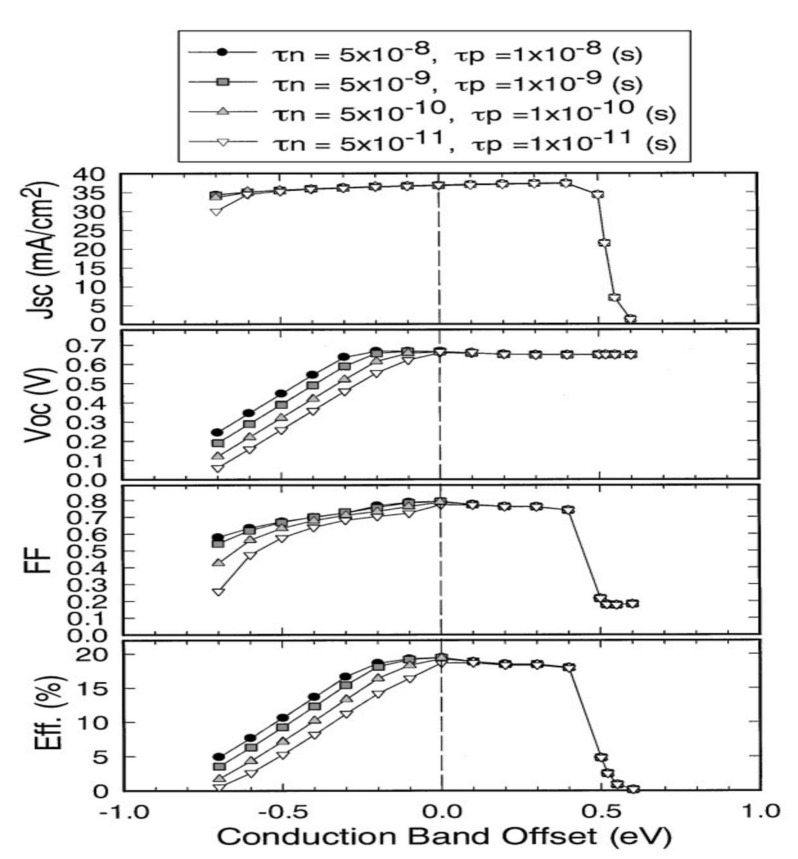
Evolution of a CIGS cell’s performance with the CBO value at the CIGS/CdS interface. τn and τp correspond to the lifetimes of electrons and holes Reprinted with permission from Ref. [21]. Copyright 2001 Elsevier.

Minemoto et al. have simulated and studied, by the finite element method, the influence of the CBO value on the performance of photovoltaic devices (Figure 4) [21].

Only devices with CBOs between 0 and 0.4 eV show good photovoltaic performance. A CBO greater than 0.4 eV leads to the formation of an energy barrier that is too high for the photogenerated electrons in the CIGS to cross. The life of the carriers is significantly reduced when the CBO value is negative. A CdS-free photovoltaic cell, i.e., with a CIGS/ZnO interface, has a negative CBO of −0.2 eV. Thus, by avoiding a negative discontinuity between the CIGS and the ZnO, the buffer layer allows the optimal alignment of the conduction bands. Historically, the buffer layer consists of chemical bath deposition (CBD) cadmium sulfide (CdS). Its advantages and disadvantages are detailed in the following section.

#### 2.3.2. The Layer of CdS by Chemical Bath

##### The Benefits of the CdS

The advantages of this buffer layer are as much related to the material as to the deposition technique used. Indeed, deposition by chemical bath makes it possible to obtain epitaxial growth of the CdS on the CIGS and reduce the defects at the interface, making it possible to minimize the recombinations of carriers [22,23]. Abu-Ras et al. compared cells with a CdS buffer layer deposited by chemical bath and evaporation [23]. If, in both cases, the diffusion of CIGS copper to CdS is observed, the interface between the absorber and the buffer layer is steeper for the evaporation-deposited CdS and has a higher defect density. Moreover, in a chemical bath preparation, the presence of ammonia in the solution makes it possible, prior to the formation of the CdS, to clean the surface of the CIGS by removing the oxides formed when the absorber is vented [24]. Moreover, the doping of the surface of the CIGS by the metal elements of the buffer layer (Cd in the case of CdS and Zn for Zn(O,S)) makes it possible to create an inversion of the conductivity type of the absorber (transition from a p-type in volume to an n-type in surface) and to bury the PN junction within the CIGS [25,26]. Since the electrical junction is separated from the physical junction, interface recombinations are minimized.

##### The Disadvantages of the CdS

The CdS buffer layer deposited by the chemical bath is associated with the best performance of CIGS cells; it nevertheless has several disadvantages. First of all, from an environmental point of view, since cadmium is a carcinogenic element, its recycling presents a potential danger [27]. Moreover, given the low value of the bandgap energy of the CdS (2.4 eV), a fraction of the photons is absorbed in the buffer layer before reaching the CIGS. Thus, all carriers generated in the CdS are not collected. Finally, from an industrial point of view, the deposition of the CdS buffer layer by the sulfur chemical bath has two significant drawbacks: the vacuum breaking in the production line (all other cell layers are deposited under vacuum) and the need for toxic waste management. Given these drawbacks, numerous studies have been carried out on alternative materials to the CdS buffer layer and their preparation by vacuum deposition methods.

#### 2.3.3. Alternative Materials to CdS

Since 1992 and the use of ZnS as a buffer layer [24,28], many researchers have been working on developing the CIGS cell without the use of cadmium. However, buffer layers that are alternative to the CdS must fulfill many specificities:

- A bandgap greater than the bandgap of CdS (2.4 eV) is suitable to maximize photon absorption in CIGS;
- Optimal alignment of the bands of conduction between the absorber and the buffer layer (0 < CBO < 0.4 eV);
- Good agreement with the crystalline structure of the CIGS to minimize interface defects;
- Higher doping of the buffer layer than that of the absorber surface to maintain the Space Charge Zone (SCZ) in the absorber;
- Low electrical resistivity—in the opposite case, the buffer layer will have to be deposited with a small thickness;
- A deposition technique compatible with an online method.

**Figure 5:**
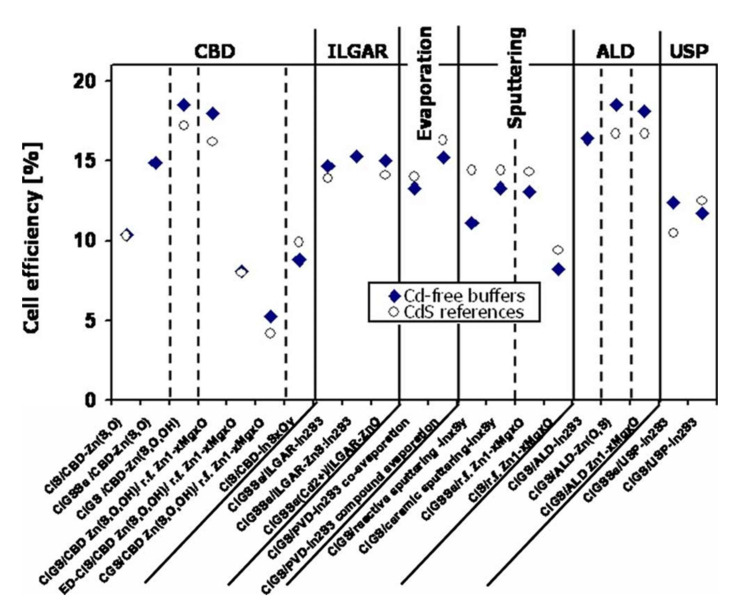
Better performance of cadmium-free photovoltaic cells as a function of buffer layer deposition methods. Comparison with cells containing cadmium Reprinted with permission from Ref. [29]. Copyright 2010 JOHN WILEY and SONS.

Numerous studies have been carried out on replacing the CdS buffer layer in CIGS cells with materials based on zinc or indium. In 2010, Naghavi et al. identified the different buffer layers and the deposit methods used to make them [29]. The best performances are presented and compared with cells with CdS in Figure 5, resulting from this review and updated with more recent published data. The best cell performance with alternative buffer layers is obtained for indium-based materials such as In_2_S_3_ or zinc such as Zn(O,S), ZnMgO, Zn(S, O, OH). These buffer layers can be deposited by chemical methods such as chemical bath (CBD), Ion Layer Gaseous Reaction (ILGAR, which is a sequential chemical, cyclic deposition), or physical processes such as evaporation, spraying, or atomic layer deposition (ALD).

The main disadvantage of In_2_S_3_ is that indium is a rare element, which leads to potentially higher production costs. In Figure 5, it can be noted that solar cells with or without cadmium have close record performances. Not all these record-breaking values are directly comparable. Indeed, several absorbers and several chemical compositions are considered in this review (CIS or CIGS). However, we can note lower performance for CIS or CGS absorbers compared to CIGS absorbers. The record yield of 22.6%, held by ZSW, is obtained with a buffer layer of CdS deposited by CBD [1]. This group regularly improves (several times a year) the record yield of CIGS cells made. Solar Frontier, currently the world’s only producer of CIGS, produces CIGS-based cells deposited by sputtering metal elements and annealing selenization. The reported (uncertified) records are 22.8% with a CdS buffer layer and 22.0% with a Zn(O, S, OH) buffer layer deposited by a chemical bath [30]. To date, CIGS cells with Zn(O,S) x as a buffer layer have achieved record yields greater than 18%, regardless of the deposit method used. The record yield is 18.7% for ALD [31] and 19.7% for CBD [32]. Note also the recent record (2014) of 18.3% obtained for a Zn(O,S) buffer layer deposited by the non-reactive sputtering of a ternary target of Zn(O,S), a record held by Klenk et al. [33].

Although the record yields obtained for solar cells comprising a Zn(O,S) buffer layer are close, the spraying method has the significant advantage of being compatible with an online industrialization process on substrates with a large area. Several techniques for obtaining the Zn(O,S) layer by spraying are listed in the literature.

- The non-reactive sputtering of a ternary target

The target used already contains all the elements: zinc, sulfur, phosphorus, and oxygen. This method has the advantage of being used in an inert atmosphere. On the other hand, the window of accessible chemical composition will be weak because it is close to that of the chosen target; this is the main disadvantage of this variant of the deposition method. Nevertheless, this combination now offers the best returns (18.3%, Klenk et al. [33]).

- Co-sputtering of ZnS and ZnO targets

This second variant of the sputtering technique is also carried out in an inert atmosphere with the co-sputtering of two targets of ZnS and ZnO. The power applied to each of the targets allows the final chemical composition of the film to be controlled across the full range of chemical composition, i.e., ZnO (zero power on the ZnS target) to ZnS (zero power on the ZnO target). The experimental development of this technique is more delicate than spraying a single target with a more consistent parametric study, especially to identify competition phenomena or positive synergy in terms of co-spraying. In addition, from an industrial point of view, the need for a second power generator generates a higher cost. Buffière et al. [34] reported a maximum yield of 8%.

- The reactive sputtering (O) of a ZnS_2_ target

This variant allows control of the final chemical composition of Zn(O,S) films in a wide range thanks to the oxygen flow control. Grimm et al. [35] hold a record yield of 13.7% in this configuration.

- The reactive sputtering (H_2_S) of a ZnO target

A final variant concerns the reactive sputtering of a ZnO target in argon and H_2_S plasma. It is not listed in the literature today, probably because of the necessary security for the use of H_2_S gas. The range of chemical composition of ZnO_1−x_S_x_ films is important. The resulting films can have a wide variety of properties that need to be controlled. The following part describes the variation of the properties of the Zn(O,S), ZnS, and ZnO films deposited by spraying with the experimental parameters (pressure and depositing power) and their chemical compositions.

#### 2.3.4. The Properties of ZnS and ZnO Films

The properties of the thin films deposited by sputtering depend on the experimental conditions used for their production. The working pressure and the power density applied to the target can change them to the first order. Table 1 summarizes some literature results concerning the influence of these two parameters on the properties of ZnO and ZnS films.

**Figure 6:**
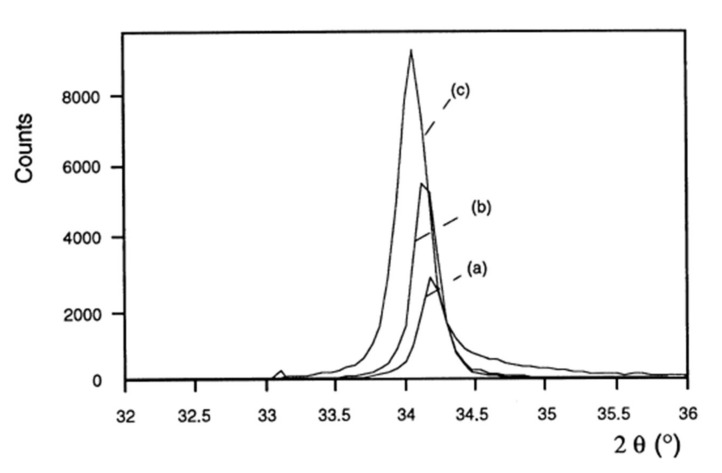
X-ray diffraction patterns of ZnO films deposited with different powers on glass substrates (oxygen partial pressure 0.01 Pa, total pressure 1 Pa): (**a**) 0.38 W/cm²; (**b**) 0.63 W/cm²; (**c**) 1.27 W/cm² Reprinted with permission from Ref. [36]. Copyright 1999 Elsevier.

No study on the influence of pressure or power on the properties of intermediate-composition ZnO_1−x_S_x_ films has been recorded. The film deposition conditions influence their crystalline and optical properties. Figure 6 shows the influence of power on the crystalline quality of ZnO films [36]. The positions of the diffraction peaks are also shifted towards the small angles, suggesting a modification of the stresses in the material. The films are more homogeneous and denser [38]. An increase in the power results in an increase in the intensity of the diffraction peaks corresponding to the orientations (002) and (004) when the material is crystallized in a hexagonal Wurtzite structure or at the orientations (111) and (220) when the crystalline mesh is cubic sphalerite [39]. This increase is accompanied by the refinement of the diffraction peaks, and therefore an increase in the size of the crystallites (according to Scherrer’s law [40]). Therefore, the crystalline quality of the films is improved for a high power density. These observations are consistent with an increase in the high power sputtering rate, which generates a more significant effect of ion bombardment on the surface of the film being deposited.

**Figure 7:**
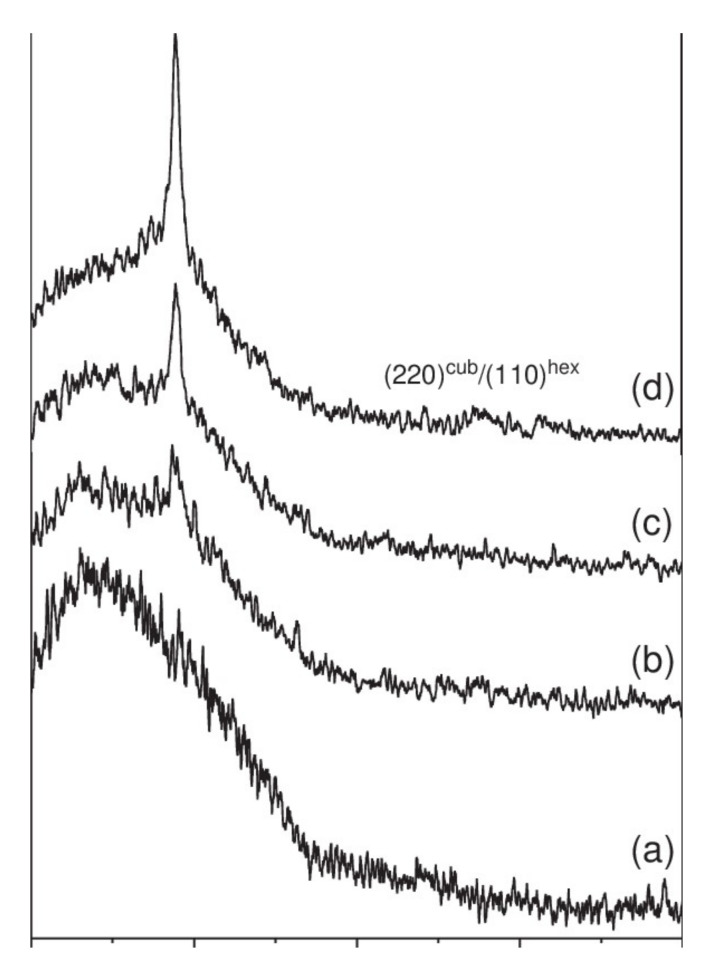
X-ray diffraction patterns of ZnS thin films deposited on glass substrates at different solution pH, (**a**) 11.5, (**b**) 10.99, (**c**) 10.31 and (**d**) 10. Reprinted with permission from Ref. [38]. Copyright 2006 Elsevier.

Nevertheless, making deposits of ZnO or ZnS at too high a power impairs the crystalline quality of the films. Thus, Hua et al. have observed the degradation of the crystalline properties of films above 15 W/cm² [38]. Indeed, if the pulverized species have too much energy or high density, too much bombardment of the film by the sputtered ions and species can lead to excessive densification and re-spraying of the deposited particles, thus leading to the degradation of the crystalline quality or amorphization of the deposited film. Optimal power must therefore be found during a parametric study. At the same time, the optical properties are little influenced by the power, with a slight decrease in the transmittance of the deposited films. Bachari et al. explain this result by the densification of the structure of the films, which would favor the reflection of the incident radiation [36]. The power applied to the target does not significantly change the energy of the optical gap, which is between 3.3 and 3.5 eV, according to the studies identified [38,39]. Figure 7 shows the diffraction patterns of ZnS films deposited at different pressures (range 0.2 to 1.5 Pa) [38]. In this study, the films have a single diffraction peak corresponding to a ZnS orientation along the (111) axis. With the pressure decrease from 1.5 to 0.2 Pa, the diffraction peak becomes more intense, and there is an increase in the size of the crystallites (Scherrer’s formula). Thus, reducing the deposition pressure of the films makes it possible to improve the crystalline quality thereof.

In the literature, differences of opinion are recorded as to the influence of pressure on the optical properties of ZnS and ZnO films. Thus, Bachari et al. observe an increase in the optical transmittance of ZnO films with an increase in pressure (range 0.25 to 6 Pa), related to the kinetic energy variation of the particles sprayed [36]. On the other hand, for ZnS films, Hua et al. do not show an evident change in transmittance with the deposition pressure in a lower pressure range (0.5 to 1.2 Pa) [38]. Nonetheless, for ZnS films, Zhang et al. observe better transmittance for a deposition pressure of 0.7 Pa but a different power density [37]. Nevertheless, the various authors agree on obtaining superior transmittance when the films have good homogeneity, few defects, and a decrease in the value of the forbidden band energy when the pressure increases. The variation of the deposition pressure and/or the power (power density) modifies the properties of the ZnO and ZnS films:

- High power generates higher crystalline quality.
- A low deposition pressure makes it possible to achieve a larger optical gap. Thus, it is necessary to precisely identify the conditions of deposits compatible with obtaining films having the desired qualities. If pressure and power influence the characteristics of ZnO and ZnS films, the chemical composition modifies their properties in a preponderant way.

#### 2.3.5. Range of Variation Properties of ZnO_x_S_1−x_ Films

The structural, physicochemical, and functional properties of ZnO_x_S_1−x_-based buffer layers strongly depend on their chemical composition. Table 2 lists the results of several studies of the literature on the influence of the chemical composition on the properties of thin films Zn(O,S). The films are obtained by reactive sputtering (Ar/O_2_) either of a ZnS target or by co-sputtering two ZnS and ZnO targets and following the studies on different substrates: SLG glass, quartz, sapphire, or CIGS. Since quartz, sapphire, and CIGS are crystallized, Zn(O,S) crystallization on these substrates is favored. They also have better resistance to temperature than the SLG glass substrate (whose limit of use is around 550 °C).

In the case of reactive sputtering (Ar + O_2_), the film’s chemical composition is modified by varying the oxygen flow rate during spraying, i.e., the proportion of oxygen in the gas phase. For co-sputtering, changing the powers applied to each of the ZnS and ZnO targets changes the film’s chemical composition.

##### The Chemical Composition of ZnO_x_S_1−x_ Films 

**Figure 8:**
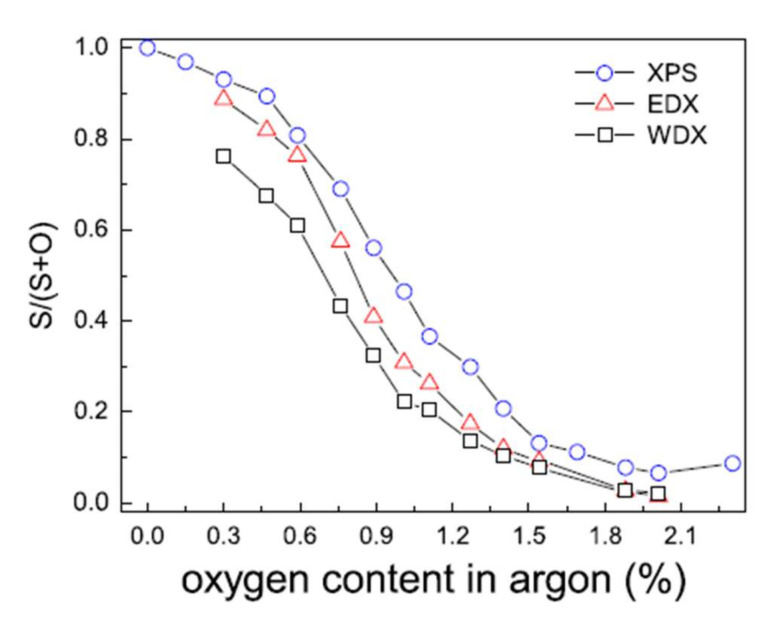
Evolution of the S/(S + O) ratio of Zn(O,S) films determined by XPS, WDS, and EDX as a function of the proportion of oxygen in the gas phase Reprinted with permission from Ref. [43]. Copyright 2011 Elsevier.

The reactive sputtering (Ar + O_2_) studies of a ZnS target have shown that as soon as oxygen is added to the gas phase, it is substituted for sulfur, leading to a mixed compound Zn(O,S) for very low oxygen flow rates [16,43]: less than 2% in the gas phase in the Grimm study (Figure 8).

Moreover, the minor variation between the surface chemical composition determined by XPS and by volume obtained by EDX and WDX is indicative of the suitable chemical homogeneity of the films. Fine control of the oxygen flow rate is necessary to control the final chemical composition of the deposited films. The authors agree on the difficulty of obtaining ZnS films free of oxygen [44].

##### The Crystalline Characteristics of ZnO_x_S_1−x_ Films 

**Figure 9:**
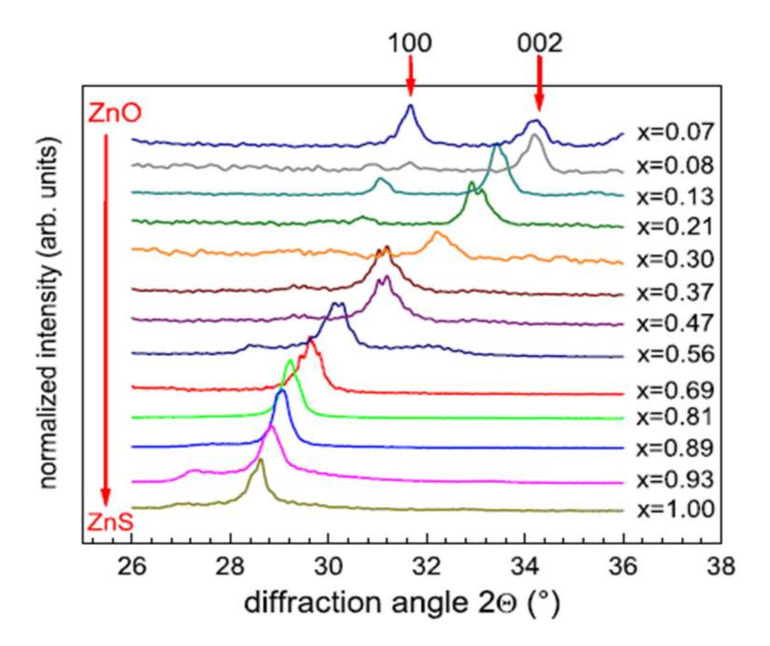
Evolution of DRX diagrams of a ZnO_1−x_S_x_ film deposited on quartz substrates at 200 °C with the chemical composition Reprinted with permission from Ref. [43]. Copyright 2011 Elsevier.

The variations in the chemical composition of the films have direct effects on the crystalline characteristics. Thus, the diffraction peak obtained at 28.5° by X-Ray Diffraction (XRD) is associated with the ZnS material without oxygen. The crystalline structure (cubic or hexagonal) is challenging to identify because of the coincidence of the diffraction peaks associated with the orientations (111) of the cubic structure and (002) of the hexagonal structure. Meyer et al. [43] remove the ambiguity by analyzing their films by grazing incidence φ-scan and reveal a hexagonal wurtzite structure for Zn(O,S) films deposited on quartz substrates at 200 °C. In Figure 9, we observe the shift of the diffraction peak corresponding to the orientation (002) towards the large angles with the increase in the oxygen content, associated with a linear decrease in the interplanar distance, in good agreement with the law of Vegard [45]. At high oxygen flow, the diffraction peaks correspond to hexagonal ZnO (wurtzite). The addition of oxygen in the gas phase thus leads to the substitution of sulfur by oxygen in the ZnS cell, forming a Zn(O,S) structure. 

Using a film consisting solely of ZnO high oxygen flow, in this study, the authors observe a continuous evolution from pure ZnS films to pure ZnO films.

These results were obtained for films deposited by heating the substrate (200 °C). For deposits made without intentional heating of the substrate [42], the decrease in the mesh parameter with the increase in the oxygen content is accompanied by a decrease in the intensity of the diffraction peak, which disappears completely with high percentages of oxygen. In addition, Choi et al. [41] obtain crystallized films (deposits at 164 °C) when the sulfur or oxygen content is high and low crystalline quality for the intermediate compositions [41]. Under the experimental conditions of their study, a thermal contribution is necessary (temperature of around 200 °C) to obtain well-crystallized ZnO_x_S_1−x_ films over the whole range of chemical composition. The surface morphology of Zn(O,S) films [41,42] is granular, dense, and uniform, with grains smaller than 50 nm, which decreases with increasing oxygen content. For high oxygen content, their films have surface cracks and are amorphous (absence of X-ray diffraction peaks). 

**Figure 10:**
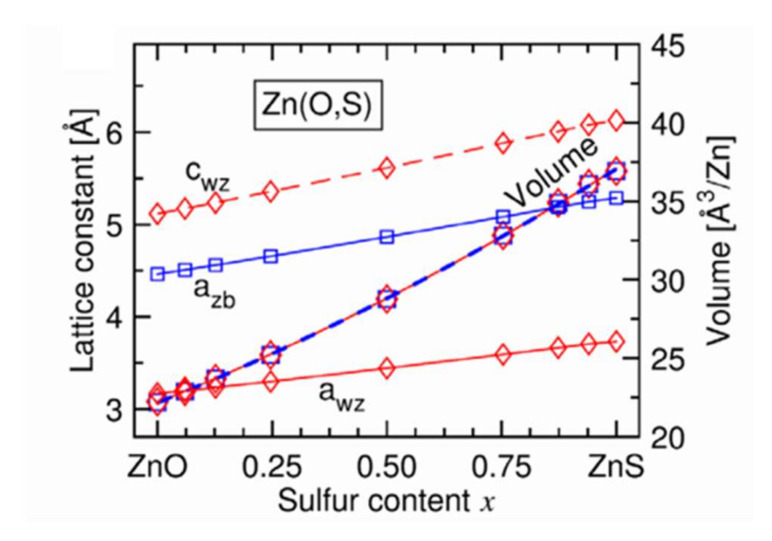
Evolution of mesh parameters and mesh size for wurtzite structure (diamond) and cubic (square) structure of Zn(O,S) films with sulfur content Reprinted with permission from Ref. [37]. Copyright 2016 AIP Publishing.

In another study, Baldissera and Persson [37] carried out structural modeling of Zn(O,S) films by Density Functional Theory (DFT) calculations. Figure 10 [37] presents the evolution of the mesh parameters a and c and the crystal mesh volume with the sulfur content for a hexagonal wurtzite structure. This evolution is linear over the whole range of composition, in good agreement with the law of Vegard. In addition, the c/a ratio increases with the sulfur content and is most significant for films having a sulfur content x of between 0.3 and 0.7 (not shown here). We also observe (Figure 10) a decrease in the volume of the elemental mesh when the chemical composition of the films passes from ZnS to ZnO, which is explained by the smaller volume of an oxygen atom. This figure shows that the sizes of the elemental mesh of the cubic zincite and hexagonal wurtzite structures are identical regardless of the chemical composition of the Zn(O,S) films.

**Figure 11:**
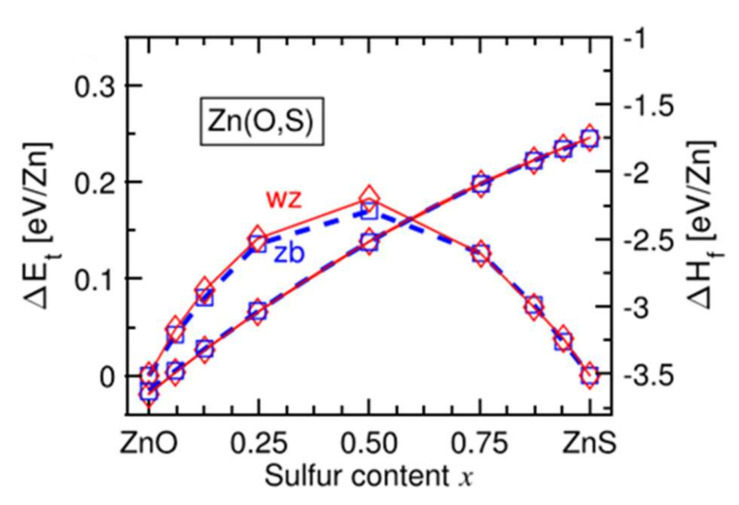
Evolution of the energies ΔE and ΔH for the wurtzite structure (denoted wz in red) and the cubic structure (denoted zb in blue) of the Zn(O,S) films with the sulfur content Reprinted with permission from Ref. [37]. Copyright 2016 AIP Publishing.

Figure 11 [37] shows that in this range of chemical composition, 0.3 < x < 0.7, the formation enthalpy of the cubic phase becomes weaker than that of the hexagonal wurtzite phase. In intermediate compositions, there is competition between the two crystalline phases. This competition can lead to the simultaneous existence of the two phases in the form of domains [37]. Under different experimental conditions, the coexistence of phases is not observed, and the films are amorphous in this composition range.

##### The Optical Properties of ZnOS_1−x_ Films

**Figure 12:**
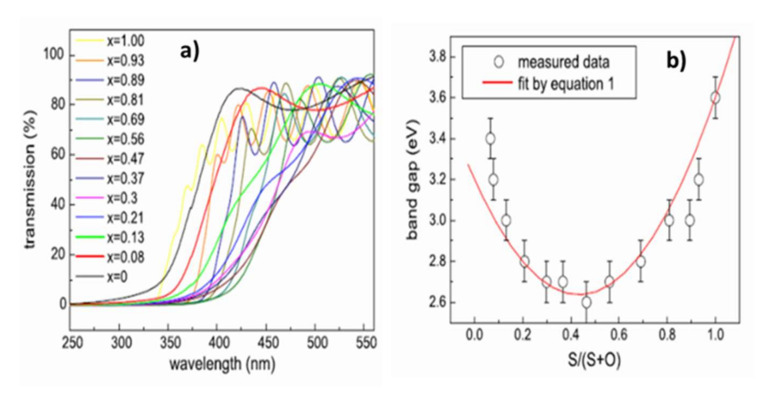
(**a**) Optical transmittance spectra by UV–visible spectrometry of films deposited on quartz substrates at 200 °C. (**b**) Variation in bandgap energy of ZnO_1−x_S_x_ films deposited on ZnO_1−x_S_x_ quartz substrates at 200 °C with the chemical composition Reprinted with permission from Ref. [43]. Copyright 2011 Elsevier.

Variations in the chemical composition of ZnO_1−x_S_x_ films also modify their optical properties. The optical transmittance spectra obtained by UV–visible spectrometry in Figure 12a shows that if all the films have a mean transmittance in the visible range of around 70%, the cutoff wavelength varies with the chemical composition. There is thus a shift towards the long wavelengths of the cut-off wavelength with the incorporation of oxygen into the films and a return from the intermediate compositions (ZnO_1−x_S_x_ with x ≈ 0, 5). The measurement of the absorption coefficient α from the transmittance and reflectance spectra allows the determination of the forbidden band energy of the material. The extrapolation to 0 of the function (αE)² = f(E) gives the value of the optical gap of the semiconductor with a direct forbidden band (the function is written (αE)^1/2^ = f(E) in the case of indirect transition).

The variation in bandgap energy is therefore correlated with the chemical composition (Figure 12b) and can be described by Equation (1):

Eg(x) = xEg(ZnS) + (1−X)Eg(ZnO) − b(1−X)X(1)

where Eg(ZnS) and Eg(ZnO) are 3.6 and 3.2 eV, respectively, and the Bowing factor b, which is an adjustment factor, is obtained by deduction (b ≈ 3 eV [43,45]). The Bowing factor is a constant, taking into account the non-linearity of the variation in bandgap energy with the chemical composition. Thus, once the pressure and the working power have been set, it is necessary to control the chemical composition of the ZnO_1−x_S_x_ films in order to be able to control their optical properties and best meet the specifications. Depending on the range of chemical compositions selected, the ZnO_1−x_S_x_ films have a larger bandgap than the CdS and optimal alignment of the conduction bands between the absorber and buffer layer (0 < CBO < 0.4 eV [47]). The films are transparent with average transmittances higher than 70% in the visible range. To our knowledge, no value of electrical resistivity of the films is recorded in the literature. If the ZnO_1−x_S_x_ films are too resistive, they must be deposited in a very thin layer to let the current flow through the tunnel effect. In the literature, no study has investigated the importance of the crystalline quality of the buffer layer or its crystallization in a hexagonal rather than a cubic structure (or vice versa).

### 2.4. Effects of Alkali Metal Halide Postdeposition Treatment

In order to improve CISe-based photovoltaic device performance, alkali metal doping is essential. Since the early 1990s [48], a variety of alkali metal doping control methods have been considered, in addition to alkali-containing glass substrates. These include using NaF or other alkali halide precursors on a Mo back contact layer [49,50], MoNa compound back contacts [51,52], or a sputtered soda–lime glass thin layer on the substrate [53,54] with post-deposition treatment (PDT) [55]. PDT using heavy alkali metal halides such as KF, RbF, and CsF has significantly improved the efficiency of CISe-based photovoltaic devices [56,57]. PDT is now one of the most promising alkali metal doping controls. Beyond the effects of alkali metals diffused from the substrate side, PDT methods using relatively heavy alkali metals likely modify the film surface (device interface), resulting in improved photovoltaic performance [58,59]. PDT effects can be caused by alkali metal compound phases such as KInSe_2_ and RbInSe_2_ [60,61]. It depends on the alkali metal species and CISe-based film properties, such as the elemental composition and surface conditions. Depending on the experimental conditions, PDT can improve or degrade device performance [62,63]. Li doping in CIGSe films and devices has minimal benefits compared to doping with heavier alkali metals such as Na, K, Rb, and Cs, which have significant benefits [64]. The beneficial effect of Na on improving photovoltaic performance has been proven [48,49,50,51,52,53,54,55]. However, the highest photovoltaic efficiency reported to date was achieved with Cs treatment of a CIGSe photoabsorber layer [56]. Control of Cu-deficient phases present on the CIGSe film surface and alkali halide supply appropriate to the surface condition are suggested to obtain beneficial effects with PDT [65]. The degree of Cu deficiency as indicated by the [Cu]/([Ga] + [In]) (CGI) and group III elemental composition ([Ga]/([Ga] + [In]) (GGI) and their fluctuations may vary depending on the CIGSe-based films prepared in each laboratory. In RbF-PDT, a CDL on the CIGSe film surface significantly affects the PDT results, resulting in changes in photovoltaic device parameters and photovoltaic performance. For example, Shogo et al. showed the photovoltaic parameter variations observed with RbF-PDT on CIGSe films grown with a high In content (GGI 0.03) CDL surface [66]. Increasing the CDL thickness (blue boxes) decreased the open circuit voltage (VOC), fill factor (FF), and concomitant photovoltaic solar cell efficiency (PVSC) [67,68]. The device performance improved with RbF-PDT when the CDL was thick enough but degraded when the CDL was absent or very thin. The literature shows a similar trend for KF-PDT [58]. RbF-PDT on a CIGSe film without a surface CDL increased interfacial recombination [55]. These findings suggest that a thick CDL is required to achieve beneficial PDT effects on the device interface.

### 2.5. The Rear Contact

The rear contact or back electrode has the primary function of collecting the holes generated by the absorption of photons and the creation of electron–hole pairs. Several conditions must be met to obtain good-quality back contact. This layer serves as a substrate for the deposition of the absorber, the buffer layer, and the window layer. It must therefore withstand the conditions of deposit and any post-deposit treatments, such as annealing. The rear contact must also have low electrical resistivity (less than 5 × 10^−5^ Ω·cm) and good optical reflectivity to minimize optical losses.

#### 2.5.1. The Role of the Back Contact

The literature describes many comparative studies on the use of various materials. Thus, tungsten, chromium, tantalum, niobium, vanadium, titanium, manganese, platinum, gold, silver, copper, and TCOs have been tested and compared to molybdenum as a rear material contact [66]. In their comparative study, Orgassa et al. observe that titanium, chromium, manganese, and vanadium react with selenium during CIGS deposition. Chemical reactions consume titanium and manganese. Tungsten, molybdenum, tantalum, and niobium are less degraded during the growth of the CIGSe absorber and do not influence the granular morphology of the CIGS film [67]. The formation of a selenium layer at the interface between the back contact and the CIGS has been observed for molybdenum, tantalum, and niobium [67]. This layer makes it possible to form an ohmic contact between the absorber and the rear. It makes it possible to extract the majority of carriers as well as possible. The other components of the CIGS can also react with the rear contact. Nakada et al. made photovoltaic cells using TCOs as the back contact [68]. They studied cell structures with AZO, tin oxide (ITO), and fluorine-doped tin oxide (SnO_2_:F) as back contacts on which CIGS was deposited by co-evaporation between 450 and 550 °C. These devices display poor performance due to the formation of a resistive layer when depositing CIGS at high temperatures. The reported yields are 0.04% for AZO, 9.6% for ITO, and 1.3% for SnO_2_:F.

#### 2.5.2. The Choice of Molybdenum 

**Figure 13:**
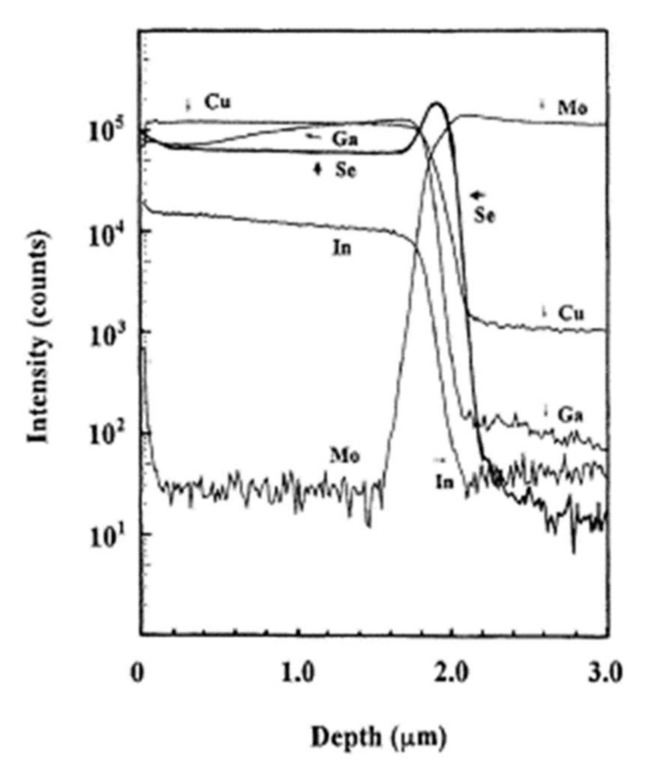
Profile of the deep chemical composition of CIGS and Mo layers by SIMS analysis Reprinted with permission from Ref. [9]. Copyright 2011 Elsevier.

Among the materials tested, only molybdenum optimally satisfies the prerequisites mentioned in the Introduction [68]. In addition, the ohmic nature of the contact with the CIGS material is ensured by the intermediate layer of MoSe_2_, which forms at the interface during the deposition and minimizes the recombinations between the carriers. In 2001, the team of Wada et al. published their work on the study of the interface between the CIGS absorber deposited by co-evaporation and the rear contact of molybdenum deposited by spraying [9]. The chemistry analyses by Secondary Ion Mass Spectroscopy (SIMS) revealed a rich selenium interface. Figure 13 shows the chemical composition profile of the CIGS and Mo layers obtained by SIMS analysis.

**Figure 14:**
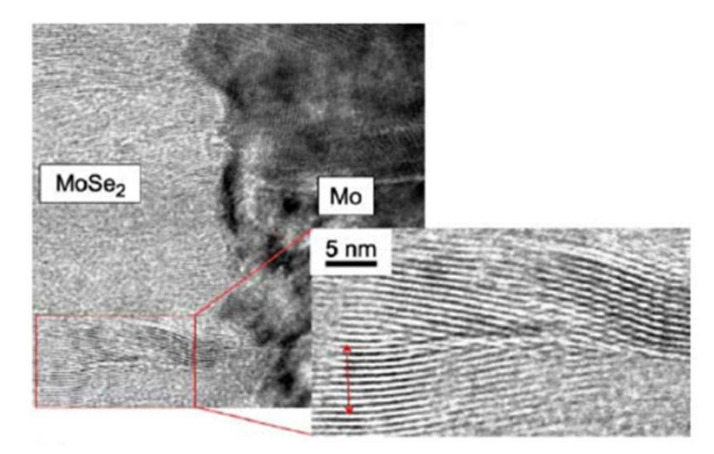
TEM images of the Mo–MoSe_2_ interface Reprinted with permission from Ref. [70]. Copyright 2005 Elsevier.

**Figure 15:**
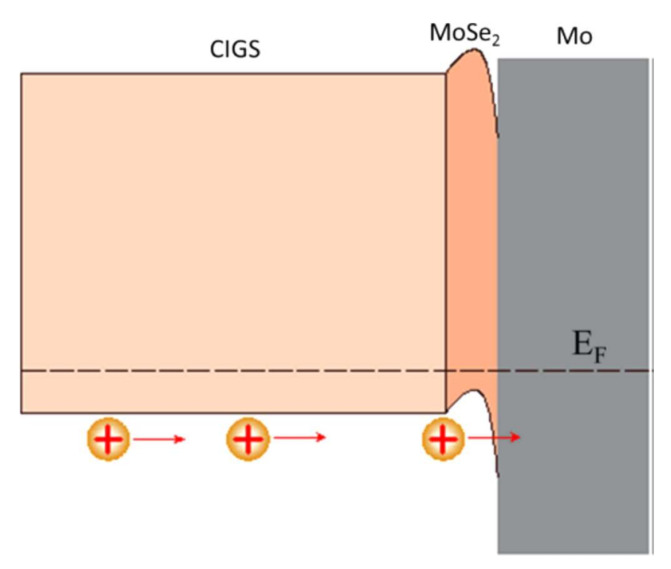
Electronic structure of the interface between molybdenum and CIGS Reprinted with permission from Ref. [69]. Copyright 2017 American chemical society.

At the interface, the intensity of the selenium shows a peak, revealing selenium accumulation, consistent with the presence of the MoSe_2_ interface layer. The TEM observations revealed a hexagonal structure according to orientations (100) and (110) and a 150 nm MoSe_2_ layer, with an axis c perpendicular to the growth axis, parallel to the surface of the Mo (Figure 14). The formation of this layer is known in the literature to facilitate the diffusion of sodium from the substrate to the absorber. Nevertheless, this interfacial layer MoSe_2_ is sometimes at the origin of poorer adhesion of the CIGS film to Mo. Moreover, the presence of this layer makes it possible to have an ohmic contact between the CIGS and Mo. The value of the MoSe_2_ gap (1.4 eV) generates a band curvature that repels electrons and effectively collects holes (Figure 15) [69].

During selenization annealing after deposition (by electrodeposition or sputtering) or co-evaporation, the reaction of selenium with molybdenum is activated with temperature. As a result, it can lead to a thick layer of MoSe_2_, reducing Mo’s thickness and deteriorating the cell’s performance. During his thesis, Aurélien Duchatelet [71] showed that it was possible to control the thickness of this layer of MoSe_2_ by prior oxidation of molybdenum, which then slows selenization. Furthermore, this work shows that the use of MoO_2_/Mo/glass substrate makes it possible to control MoSe_2_ formation thanks to the blocking effect of MoO_2_ oxide.

#### 2.5.3. The Properties of Molybdenum

DC magnetron sputtering is the most widely used technique for molybdenum deposition. Table 3 presents some selected results from the literature and illustrates the influence of the experimental parameters on the properties of Mo films. Pressure is the deposition parameter most often studied because it directly influences the electrical resistivity of films. The higher the deposition pressure, the thinner the molybdenum layer while being resistive [72,73,74,75]. This observation is explained by the microscopic structure of molybdenum and by its crystalline properties. In fact, at low pressure, molybdenum is better crystallized in its centered cubic structure, which favors better electrical conductivity [74]. Characterizations by electron microscopy have shown that molybdenum has a columnar microstructure. High deposition pressure intensifies this columnar appearance and increases the intercolumnar structure, which becomes dendritic and amorphous. The columns consist of unoxidized molybdenum, while the intercolumn space comprises molybdenum oxide and sodium [76]. Pressure, therefore, influences the microstructure of molybdenum. Although, at low pressure, there is a slight collision in the plasma between species, the average free path of the species is equivalent to the distance between the target and the substrate (of the order of ten cm) [77]. The molybdenum atoms arrive on the substrate with high kinetic energy, which favors the crystallization of the material. Poor adhesion of the molybdenum film has been observed when it is deposited at very low pressure. At higher pressure, the molybdenum atoms collide in the plasma and arrive on the substrate with lower kinetic energy, which leads to a somewhat amorphous material. In addition, at high pressure, molybdenum adheres better to the substrate.

**Figure 16:**
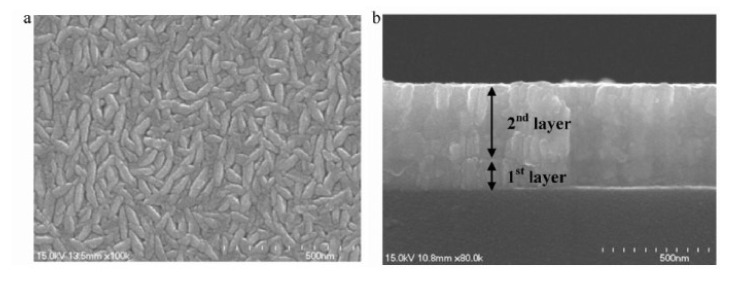
SEM images of a molybdenum bilayer: (**a**) top view and (**b**) cross-section Reprinted with permission from Ref. [78]. Copyright 2011 Elsevier.

A method was developed by Scofield et al. to obtain weakly resistive molybdenum films with good adhesion to the glass substrate [75]. Their study highlights the effect of pressure on film properties (adhesion, crystallinity, and resistivity). A slight variation in the resistivity (approximately 10 × 10^−6^ Ω·cm) is observed between 0.2 and 2 mTorr (i.e., between 2.66 × 10^−2^ Pa and 2.66 × 10^−1^ Pa). Beyond 2 mTorr, the films are more resistive, and all pass the adhesion test to the “scotch test”. From these observations, the molybdenum is deposited as a bilayer, with a first layer at 10 mTorr (1.33 Pa) for adhesion and a second deposited at 1 mTorr (0.13 Pa) for low resistivity. Good adhesion is obtained for all bilayer films with resistivities between 12 and 15 × 10^−6^ Ω·cm, compatible with the use of −6 as a back contact. Other authors have taken up this type of bilayer structure, as illustrated in Figure 16 [78].

Molybdenum is, therefore, the electrode material that best meets the specifications with:

- Resistance to the deposition conditions of the upper layers, low electrical resistivity;
- The formation of an ohmic contact at the interface with the absorber, minimizing carrier recombination;
- A strong reflective character.

The deposition conditions, particularly the working pressure, strongly modify the properties of the molybdenum films. As a rear contact, it is advisable to deposit the molybdenum bilayer structure: a first layer promoting adhesion and a second guaranteeing low electrical resistivity.

### 2.6. Front Contact

The role of the front contact or window layer is to collect the electrons. This electrode is typically composed of intrinsic zinc oxide (ZnO) and aluminum-doped zinc oxide (AZO). These two materials must have high transparency to maximize the transmission of photons to the absorber.

#### 2.6.1. The Properties of ZnO

Zinc oxide is an n-type semiconductor. It is deposited with a small thickness of around 50 nm. Its function is to limit electronic losses [18]. A material can crystallize according to three structures:

- The cubic structure [79];
- The blended structure [80];
- The wurtzite structure [80].

The wurtzite structure is the most stable at ambient temperature and pressure; it has a hexagonal symmetry. Zinc oxide absorbs little in the visible range and has a direct optical gap close to 3.4 eV at room temperature. The resistivity of ZnO depends on the thermal treatments performed on the material and varies in the range 10^−4^–10^−9^ Ω·cm [81,82]. In addition, the electrical resistivity of ZnO can be modified by doping, introducing excess zinc atoms, or creating oxygen vacancies. Interstitial zinc defects have a greater influence on zinc oxide doping because they have donor levels closer to the conduction band [83,84]. These defects within the crystal structure constitute intrinsic doping. Zinc oxide can also be doped with the introduction of foreign elements, and it is extrinsic doping.

#### 2.6.2. The Doping of ZnO and the Interest of AZO

The intrinsic doping of ZnO is not enough to make it a conductive material, and it is necessary to introduce impurities to improve its conductivity. ZnO is a II-VI semiconductor. Aluminum, gallium, and indium with additional zinc valence (column III of the periodic table) contribute to the n-type doping of zinc oxide. Aluminum has a donor energy level closer to the conduction band of zinc oxide; it is the most used dopant. Zinc oxide doped with aluminum or AZO has crystalline and optical properties close to zinc oxide. It crystallizes in the wurtzite structure and has visible transparency more significant than 90% [85]. The resistivity of AZO is dependent on the deposition technique used. If the sputtering achieves resistivity of 10^−4^ Ω·cm [85], it is only 10^−2^ Ω·cm for AZO when sprayed [86].

**Figure 17:**
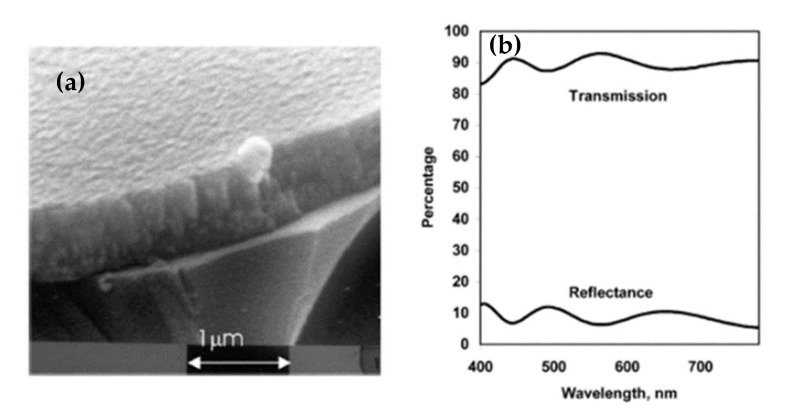
(**a**) SEM image of AZO film deposited by DC pulse spray; (**b**) Transmittance spectra of pulsed DC-deposited AZO film after annealing at 500 °C Reprinted with permission from Ref. [85]. Copyright 2003 Elsevier.

Given these results, sputtering is the most widely used technique for depositing aluminum-doped zinc oxide. For this, two types of targets can be used. On the one hand, an AZO target (doped at 2% or 3% depending on the studies) has the advantage of working with inert gas but reduces the choice as to the power supply (RF, pulsed DC, or HIPIMS). On the other hand, a zinc target is doped with aluminum, which offers the DC as an additional choice but requires working with a reactive gas, oxygen. Kelly et al. have shown interest in making films by pulsed DC spraying (described in Chapter II) to overcome the disadvantages of DC and RF [85]. As a result, they obtain very dense and defect-free films, whose SEM image is shown in Figure 17a. The morphology of the films is columnar and very regular. These films have a mean transmittance in the visible range of 90%; the transmittance and reflectance spectra are shown in Figure 17b. They are crystallized in a hexagonal structure according to the (002) orientation and have electrical conductivity of 2.7 × 10^−3^ Ω·cm.

Since Kelly et al.’s work, other groups have studied the deposition of AZO pulsed DC films. Some results are presented in Table 4. 

The films obtained have low resistivity and high average transmittances, thus meeting the set specifications. Looking at the results in Table 4, we see that the AZO film of Chung et al. has a slightly higher electrical resistivity of 1.2 × 10^−3^ Ω·cm. This film is deposited at room temperature, without intentional heating during the deposition. This minimum of resistivity in their study on the influence of pressure on the properties of AZO is obtained at 2.7 Pa. They highlight the instability of the structure of AZO when it is deposited without heating, which increases its electrical resistivity. Kar et al. have also studied the influence of the pressure on the properties of AZO in the context of deposits made by heating the substrate gate. They also observed a minimum of electrical resistivity at an intermediate pressure of 0.4 Pa, explained by the better crystalline quality of the films, which improves the mobility of the carriers. Some authors have performed AZO deposition in reactive mode by adding dihydrogen in the gas phase. The study of Li et al. shows the influence of the flow rate of H on the properties of the films. They observe better electrical properties for the films deposited in reagents than those deposited in inert gas. Hydrogen plays an essential role in the electrical properties of films by its donor function, which increases the density of carriers in films. The bibliographic study of the front contact has highlighted the interest in using aluminum-doped zinc oxide deposited by pulsed DC sputtering. 

## 3. Comparative Study of the Copper Indium Gallium Selenide (CIGS) Solar Cell with Other Solar Technologies

The primary light-absorbing material is used to characterize solar cell technologies [91]. Silicon-based photovoltaic technology has been the most widely used in commercial PV modules. Its toxicity is not an issue, and it has a great efficiency–price relationship, making it the most reliable technology due to available knowledge. However, new photovoltaic technologies have been developed that may offer significant advantages in the near future, in the sense that their efficiency has improved, and they may be easier and cheaper to manufacture [92,93]. With the development of new photovoltaic technologies over the years, it is possible to classify solar cells into four main categories known as generations, though the existence of the fourth generation is debatable [94].

The first generation includes technologies based on thick crystalline films. It includes crystalline silicon (c-Si), polycrystalline (multi-Si), and monocrystalline (mono-Si) cells, as well as wafer-based c-Si and GaAs cells used for multi-junction cells with multiple p–n junctions made of different semiconductor materials. To reduce costs associated with first-generation technology, the second generation is based on the use of thin-film technology, the advantages of which are the reduction in semiconductor material used and the lower consumption of energy during production, resulting in a cost reduction [94]. As a result, second-generation technology is usually less expensive than first-generation technology. It consists of thin-film solar cells made of amorphous silicon (a-Si), microcrystalline silicon, cadmium telluride (CdTe), and CIGS [94]. The third-generation principle is the fabrication of high-efficiency devices using second-generation thin-film deposition techniques, as well as new semiconductor architectures that span multiple energy levels or can use nanostructured or organic materials [94]. It includes perovskite cells. The fourth generation aims to improve the optoelectronic characteristics of low-cost photovoltaic panels, which are a hybrid of organic and inorganic materials [94]. Table 5 compares the cell conversion efficiency and module conversion efficiency of the most used PV technologies [95,96].

**Figure 18:**
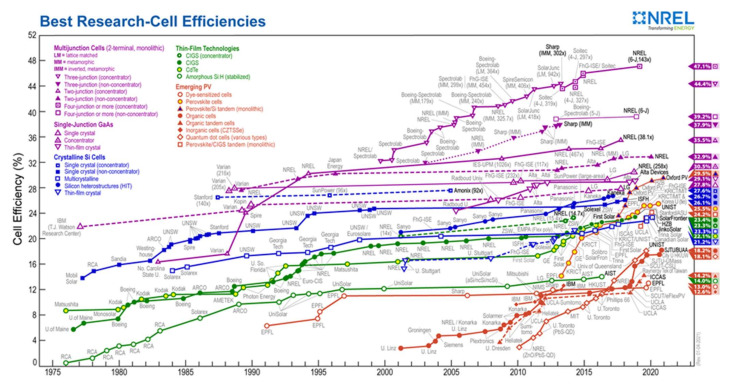
Reported timeline of research solar cell energy conversion efficiencies since 1976 (National Renewable Energy Laboratory) [97].

On the other hand, perovskite materials have complementary solar spectral absorption, bandgap tunability, ease of processing, and process compatibility with silicon and CIGS (Cu(In, Ga)Se_2_) technologies. The scientific community has recently focused on two perovskite solar cell technologies: perovskite/Si tandem and perovskite/CIGS tandem. Figure 18 shows that even though these technologies have only been studied for a few years, the efficiency of these cells is very high and rising rapidly. Oxford PV currently has the highest certified efficiency for perovskite/Si tandem cells (29.5%), followed by the Helmholtz-Zentrum Institute in Berlin (24.2%) [77]. Both mechanically stacked and monolithic tandem solar cell architectures exist. Unstacked solar cells are mechanically stacked, while monolithic cells are two-terminal devices connected in series on a single substrate. The monolithic structure is the most common and preferred, technologically and performance-wise.

Monolithic perovskite/Si tandem cells have a lot of potential to overcome the theoretical efficiency limit of single-junction silicon solar cells [98]. Perovskite/Si solar cells have a top perovskite cell and a bottom Si cell [99]. Silicon uses the red part of the solar spectrum to generate electricity, while perovskites use the blue. A tandem solar cell made of stacked silicon and perovskite can achieve efficiencies of over 30% [99]. High-efficiency monolithic tandem cells require photocurrent matching between the two subcells [99]. Optimizing the bandgap and optical density of the perovskite absorber can improve the efficiency of a perovskite/Si monolithic tandem cell. Monolithic perovskite/CIGS tandem solar cells are made of a perovskite top cell directly on a CIGSe bottom cell. In conjunction with CIGS solar cells, low-temperature semitransparent perovskite materials have reported power conversion efficiencies in excess of 20%. With complementary absorption spectra, perovskites and CIGS materials can potentially achieve PCEs of 30%. To maximize device efficiency, the interconnecting layer in tandem cells should be both electrically and optically transparent [99].

The importance of nanotechnology for improving the efficiency of solar cells is well known. High-efficiency nanotechnology-based solar cells are a potential future PV technology. Carrier collection efficiency is improved by matching the crystallite size to the carrier scattering length. By altering the nanostructure size, the bandgap may be controlled to absorb various photon energies. Individual nanostructures with homogeneous diameters below 20 nm are needed to attain these benefits at non-cryogenic temperatures. Making large arrays of nanostructures with regulated periodicity and size has proven expensive. They are inappropriate for solar applications because of their high production costs. Deposition from a colloidal particle suspension, semiconductor cluster incorporation in organic polymers, semiconductor microcrystallites in glass matrices, and strain-induced self-organized growth are examples of nonlithographic production processes. The control over nanostructure size distribution, periodicity, and semiconductor material adaptability is often lacking. Sunlight is a free energy source, but solar panels are not. However, amorphous silicon thin-film cells save money. Their decreased production costs have boosted solar energy consumption, but not enough to compete with current grid prices. To overcome this cost hurdle, researchers are now investigating nanotechnology. Nanotechnology promises to lower the prices per cell and the initial cost of a new cell type by allowing less control over the process. In addition to assisting in the usage of other promising technologies, it is being explored and developed to increase efficiency. Using nanotubes with nanoparticles or adding nanoparticles to the matrix may improve electron transport. In solar photovoltaic cells, nanoparticle interactions are well characterized. Proof-of-concept photovoltaic cells employ small perfect crystals rather than huge flawless silicon crystals. Nanowhiskers are an anti-reflective coating. It is possible to employ materials that lack this important property by adding sensitizing dyes. For several electrons per photon, quantum dots may be an enhancement over dyes. Molecular circuits that can self-power and store data via optical light might be developed. The many uses of nanotechnology make this a promising area.

## 4. Conclusions

The objective of this review was to present the latest advances for CIGS solar cells. For this, we presented the photovoltaic effect and the usual materials and the structure of the CIGS cell, namely a photovoltaic cell in which each layer is deposited by magnetron sputtering. This deposit method has the advantage of being industrialized and compatible with deposits on large surfaces.

By a bibliographic search, we have highlighted the deposition parameters playing preponderant roles in the properties of the different layers of the cell. Thus, the rear contact of the cell (the lower electrode) in molybdenum is sensitive to the deposition pressure. It should be deposited in a two-layer structure: a lower layer of thin thickness maximizing the adhesion to the glass substrate, and an upper layer of greater thickness responsible for the electrical conductivity. We saw that the front contact consisting of intrinsic zinc oxide and zinc oxide doped with aluminum and deposited by pulsed DC spraying had the best electrical performance when deposited by heating or adding hydrogen. Few parametric studies have been carried out on the Zn(O,S) buffer layer. The primary studies focus on the influence of the chemical composition and, therefore, the oxygen flow rate when the buffer layer is deposited by sputtering. If the films are generally transparent and resistive, according to their chemical compositions, the films do not have the same crystalline qualities or the same optical gap energies. Furthermore, it is mainly this optical gap value that makes them compatible for use as a buffer layer.

## Figures and Tables

**Table 1:** Selected results from the literature on the influence of working pressure and power density on the properties of ZnO and ZnS films (PW: power, PPa: pressure, Eg: gap energy optical, T: average transmittance in the visible range, D: the size of the crystallites); nominal power (unknown target size).

Target	Pressure (Pa)	Power Density (W·cm^2^)	Crystalline Properties		Optical Properties		Ref
Gas			Crystalline Quality	D (nm)	Transmission (%)	Eg (eV)	
ZnO	0.25 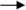 6	0.38 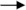 1.27	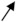 with Pw	18 < D< 46	T > 90% 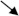 with Ppa 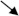 with Pw	-	[36]
Ar							
ZnS	0.1 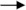 1.2	120 W	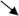 with Ppa maximum at 0.5 Pa	D < 17	T ≈ 70% maximum at 0.7 Pa	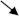 with Ppa	[37]
Ar							
ZnS	0.1 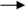 1.5	5 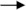 20	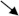 with Ppa 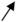 with Pw	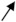 with pw 19 < D < 20	T = 80%	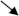 with Ppa	[38]
Ar							
ZnS	1.33	4.25 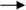 5.75	Max. at 4.6 W/cm^2^	35 < D < 70	T = 80%	Eg = 3.3 eV	[39]
Ar							

**Table 2:** Selection of results in the literature on the influence of oxygen on the properties of ZnOxS1_−_x films (Eg: optical gap energy, b: Bowing factor, T: mean transmittance in the visible range).

Technic	Experimental Parameters	Substrate		Chemical Composition	Crystalline Properties	Morphology	Optical Properties	Ref.
Target		Material	T°					
sputtering	60 W; 0.13 Pa O_2_: 0 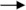 2%	Glass	164°	0.048 < O/ (S + O) < 0.82	Hexagonal structure of the mesh parameter with O	Granular surface Grains < 50 nm	-	[41]
ZnS								
sputtering	1 W/cm^2^; 1.33 Pa; O2: 0 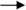 1%	Glass	No	0.2 < O/ (S + O) < 0.95 ZnO_0.05_S_0.95_ 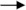 ZnO_0.71_S_0.29_	Of the mesh parameter and the intensity of O no diffractyion at the strong % O	Granular surface cracking at high% O	-	[42]
ZnS								
sputtering	2 W/cm^2^; 0.9 Pa; O_2_: 0 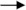 2.5%	CIGS quartz	200°	ZnS 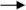 ZnO	Hexagonal structure of the mesh parameter with O	-	T≈70% 2.6 < Eg < 3.6 eV b = 3	[43]
ZnS								
sputtering	300 W; Ar/O_2_: 1.1 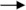 4.6%	saphir	340°	ZnO 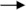 ZnO_0.6_S_0.4_	Hexagonal structure of the mesh parameter with O	crystallites size: 60–70 nm	2.6 < Eg < 3.6 eV b = 3	[44]
ZnS								[45]
sputtering	300 W			ZnS 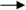 ZnO	-	-	2.7 < Eg < 3.2 eV b = 3.1	[35]
ZnS								
Co-pulverisation	PZns = 45 W; PZnO: 0 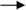 120 W	Si quartz	200°	0 < O/ (S + O) < 0.88	Hexagonal structure of the mesh parameter with O	Grains; crystallites < 120 nm	T≈80 2.7 < Eg < 3.1 eV	[46]
ZnS and ZnO								
Co-pulverisation	0.07 Pa; PznS = 45 w; PZnO: 20 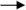 120 W			0.03 < S/ Zn < 0.90				
ZnS and ZnO								

**Table 3:** Result selections identified in the literature on the influence of experimental parameters on the properties of Mo films.

Experimental Parameters	Thickness	Crystalline Properties	Morphology	Electrical Properties	Ref.
0.026 to 0.66 Pa 200 to 300 W	300 μm	(110) Crystalline quality decrease with Ppa	-	0.4 to 6 Ω/sq 120 to 1800 × 10^−6^ Ω·cm Increase with Pw and Ppa	[72]
0.33 to 2.66 Pa	-	(110) Crystalline quality decrease with Ppa	Columnar	Increase with Ppa	[74]
0.26 to 2.6 Pa	-	-	Surface grains increment of intergrain space with Ppa	Increase with Ppa 13 to 40 × 10^−6^ Ω·cm	[73]
0.026 to 2.6 Pa	500 nm	(110) Crystalline quality decrease with Ppa	Columnar structure surface grains Density decrease with Ppa	10.8 to 250 × 10^−6^ Ω·cm	[75]
0.3 to 2 Pa	0.3 to 2 μm	-	Surface grains increment of intergrain space with Ppa made of MoO_3_	0.5 Ω/sq	[76]

**Table 4:** Selection of results identified in the literature on the influence of experimental parameters on the properties of AZO films deposited by DC pulse spraying.

Experimental Parameters	Electrical Properties	Optical Properties	Ref.
0.6 Pa, 1 W/cm^2^, 30 kHz, 280 °C, O_2_ = 15%	ρ = 3.4 × 10^−4^ Ω·cm	T = 80%	[87]
70 kHz, 230 °C, 0.13 to 2 Pa	ρ = 2.2 × 10^−3^ Ω·cm for 0.4 Pa	T = 80% Eg = 3.6 eV	[88]
30 kHz, RT, 1 to 5.3 Pa	ρ = 1.2 × 10^−4^ Ω·cm for 2.7 Pa	T = 84%	[89]
4.5 W/cm^2^, 20 kHz, RT H_2_: 0 to 20 sccm	ρ = 4.10 × 10^−4^ Ω·cm for 14 sccm	T = 85%	[90]

**Table 5:** PV technology types and efficiencies.

Solar Cell Technologies		Cell Conversion Efficiency	Module Conversion Efficiency
Crystalline	Monocrystalline silicon	27.6%	24.4%
	Microcrystalline Si	23.3%	20.4%
	Multi-junction gallium arsenide (GaAs)	47.1%	38.9%
Thin film	Cadmium telluride (CdTe)	22.1%	19%
	CIGS	23.4%	19.2%
Emerging	Perovskite	25.5%	17.9%
	Organic	18.2%	11.7%

## Data Availability

Not applicable.

## Abbreviations

AFM	Atomic Force Microscopy
ALD	Atomic Layer Deposition
AZO	Aluminum doped Zinc Oxide
CBD	Chemical Bath Deposition
CIGS	Cu(In, Ga)Se_2_
DC	Direct Current
ZSW	Zentrum für Sonnenenergie- und Wasserstoff-Forschung Baden-Württemberg
EDX	Energy dispersive X-Ray Spectrometry
EELS	Electron Energy Loss Spectroscopy
SEM	Scanning Electron Microscopy
TEM	Transmittance Electron Microscopy
PET	Polyethylene Terephthalate
PVD	Physical Vapor Deposition
RC	Cyclical report
RF	Radiofrequency
SAED	Selected Area Electron Diffraction
Sccm	Standard Cubic Centimeter per Minute (cm^3^/min)
SLG	Soda Lime Glass
TCO	Transparent Conductive Oxide
XPS	X-Ray Photoelectron Spectroscopy
XRD	X-Rays Diffraction
Zn(O,S)	zinc Oxysulfide
ZnO	Zinc-Oxyde
ZnO:Al	Aluminum Doped Zinc Oxide
ZnS	Zinc Sulfide
